# Fractionation and Characterization of Triterpenoids from *Vaccinium vitis-idaea* L. Cuticular Waxes and Their Potential as Anticancer Agents

**DOI:** 10.3390/antiox12020465

**Published:** 2023-02-12

**Authors:** Gabriele Vilkickyte, Vilma Petrikaite, Mindaugas Marksa, Liudas Ivanauskas, Valdas Jakstas, Lina Raudone

**Affiliations:** 1 Laboratory of Biopharmaceutical Research, Institute of Pharmaceutical Technologies, Lithuanian University of Health Sciences, Sukileliu Av. 13, LT-50162 Kaunas, Lithuania; 2 Laboratory of Drug Targets Histopathology, Institute of Cardiology, Lithuanian University of Health Sciences, Sukileliu Av. 13, LT-50162 Kaunas, Lithuania; 3 Department of Analytical and Toxicological Chemistry, Lithuanian University of Health Sciences, Sukileliu Av. 13, LT-50162 Kaunas, Lithuania; 4 Department of Pharmacognosy, Lithuanian University of Health Sciences, Sukileliu Av. 13, LT-50162 Kaunas, Lithuania

**Keywords:** *Vaccinium vitis*-*idaea* L., lingonberry, cuticular waxes, triterpenoids, semi-preparative fractionation, anticancer agents, cell viability, spheroids

## Abstract

Fruit and leaf cuticular waxes are valuable source materials for the isolation of triterpenoids that can be applied as natural antioxidants and anticancer agents. The present study aimed at the semi-preparative fractionation of triterpenoids from cuticular wax extracts of *Vaccinium vitis-idaea* L. (lingonberry) leaves and fruits and the evaluation of their cytotoxic potential. Qualitative and quantitative characterization of obtained extracts and triterpenoid fractions was performed using HPLC-PDA method, followed by complementary analysis by GC-MS. For each fraction, cytotoxic activities towards the human colon adenocarcinoma cell line (HT-29), malignant melanoma cell line (IGR39), clear renal carcinoma cell line (CaKi-1), and normal endothelial cells (EC) were determined using MTT assay. Furthermore, the effect of the most promising samples on cancer spheroid growth and viability was examined. This study allowed us to confirm that particular triterpenoid mixtures from lingonberry waxes may possess stronger cytotoxic activities than crude unpurified extracts. Fractions containing triterpenoid acids plus fernenol, complexes of oleanolic:ursolic acids, and erythrodiol:uvaol were found to be the most potent therapeutic candidates in the management of cancer diseases. The specificity of cuticular wax extracts of lingonberry leaves and fruits, leading to different purity and anticancer potential of obtained counterpart fractions, was also enclosed. These findings contribute to the profitable utilization of lingonberry cuticular waxes and provide considerable insights into the anticancer effects of particular triterpenoids and pharmacological interactions.

## 1. Introduction

Natural products constitute a considerable part of newly proposed drugs. Nowadays, Hippocrates’s famous quote “Let food be your medicine” has turned to plant constituents that could be used in the development of new therapeutic agents for the management and prevention of different diseases, including cancer. Phytochemical research tends to focus on finding novel anticancer phytopharmaceuticals to fulfill current needs [1,2,3]. Anticancer drug discovery is a challenging area for scientists in which pharmaceutical industries are investing heavily. Despite numerous cancer therapies, no medicine has been proven to be fully safe and effective, therefore cancer is yet one of the most devastating and life-threatening diseases throughout the world [4]. Chemotherapy multidrug resistance, high toxicity due to non-selectivity, and unfavorable adverse effects of drugs developed by synthesis have encouraged the demand of utilizing the bioactive compounds from plant resources [1,5].

Triterpenoids, distributed widely in higher plants, represent an important and abundant group of specialized plant metabolites with structural diversity in carbon skeletons and functional groups [6]. These compounds have a significant role in plant secondary metabolism and may serve as defensive constituents against herbivores and pathogens [7]. Many triterpenoids—such as ursolic, oleanolic, betulonic, betulinic, maslinic acids, and their derivatives—are highly important in pharmacology and have been characterized as powerful antioxidants and multi-targeting antimicrobial, antiviral, anti-inflammatory, immunotherapeutic, and antineoplastic agents with the most prominent anticancer effect [5,8,9]. Since oxidative stress can result in tumor initiation and promotion, triterpenoids that inhibit the overproduction of intracellular reactive oxygen species contribute significantly to chemoprevention [10]. Studies demonstrated that triterpenoids are capable to act in various stages of carcinogenesis, modulate the cell cycle, inhibit proliferation and metastasis, and induce apoptosis in malignant cancer cells [6,8,9,11]. The multifunctionality of triterpenoids makes them promising drug leads, which could serve for prototyping new anticancer drugs [12]. The first steps include the phytochemical screening of triterpenoid-rich plants; further fractionation, purification, and identification; followed by their toxicological and clinical evaluation [13].

Lingonberry (*Vaccinium vitis-idaea* L.) plant, a perennial evergreen Ericaceous dwarf shrub with edible fruits, is included in a healthy diet of the Northern Hemisphere due to its high nutritional value and richness of bioactive compounds. Data suggest that lingonberry raw materials are underutilized natural resources for developing medicines or functional food [14,15,16]. The purification of targeted compounds from lingonberry extracts is complicated by the matrix complexity and presence of notable amounts of a wide range of potentially active and non-active components that may interfere with each other [17,18]. Previous studies from our laboratory [19,20] have focused on phenolic compounds fractionation using Sephadex LH-20 and ethnopharmacological potential evaluation. One primary problem with this technique is that different groups of specialized metabolites, such as phenolics and triterpenoids cannot be purified in a single run from a crude matrix. Therefore, high waste production of potentially active compounds is generated. Moreover, the food industry produces huge amounts of by-products and industrial leftovers each year when processing fruits for juice, syrup, or jam production. The waste generated by lingonberry processing is berry “skins”, which include layers of epidermal cells and cuticle that can be utilized for the isolation of valuable compounds [21,22]. Sustainable consumption, zero waste strategies, and eco-friendly approaches for waste processing become particularly relevant when addressing economic, environmental, and social value issues [23]. One of the main lingonberry by-products, the cuticle, acts as interference between plants and the environment and is defined as a hydrophobic outermost layer, composed of waxes that are embedded in the cutin (intracuticular wax layer) or cover the outer surface as a whitish, dull, and glossy coating (epicuticular wax layer) [12,24]. Cuticular waxes have attracted many researchers’ attention as the source of non-polar or intermediate-polarity compounds, namely fatty acids, alcohols, aldehydes, ketones, alkanes, wax esters, triglycerides, polysaccharides, phenolic acids, and terpenes [24,25]. *Vaccinium* berries are particularly distinguished by the abundance of triterpenoids as the main components of cuticular waxes. Although the triterpenoid profile of lingonberry fruit cuticles has been reported earlier [22,26], no study to date has investigated wax composition of evergreen lingonberry leaves with a chief advantage of seasonal availability throughout the year [27].

Taking into account the sustainable processing of lingonberry raw materials and supporting the concept that triterpenic compounds are located mainly in cuticular waxes, triterpenoid-rich extracts from lingonberry cuticular waxes were obtained in the present study and fractionated using semi-preparative HPLC, followed by analyzing triterpenoid composition by HPLC-PDA and GC-MS techniques. Furthermore, obtained cuticular wax extracts and triterpenoid-rich fractions were screened for anticancer activity towards human colon adenocarcinoma (HT-29), malignant melanoma (IGR39), clear renal carcinoma (CaKi-1) cancer 2D- and 3D-culture models and normal endothelial cells (EC) using MTT and spheroid growth and viability assays. The extraction and fractionation technologies we employ can reduce the amount of waste and utilize other bioactive compounds as phenolics, which can be further isolated from the lingonberry matrix after extraction of triterpenoids from the cuticle. To the extent of our knowledge, this is the first comprehensive report on fractionating different groups of triterpenoids from lingonberry cuticular waxes to identify potential anticancer agents.

## 2. Materials and Methods

### 2.1. Chemicals and Reagents

Analytical-grade solvents—namely chloroform, dimethylformamide (DMF), dimethyl sulfoxide (DMSO) methanol, and acetonitrile—used for extraction, sample preparation, and chromatography were purchased from Sigma-Aldrich (Steinheim, Germany), trifluoracetic acid—from Merck (Darmstadt, Germany), and purified deionized water was produced using Milli-Q system (Millipore, Bedford, MA, USA). Authentic standards of benzoic, *trans*-cinnamic, *p*-coumaric, sinapic, ferulic, maslinic and oleanolic acids, erythrodiol, α-amyrin, β-amyrin, β-sitosterol, taraxasterol, lanosterol, and lupeol were obtained from Sigma-Aldrich; betulinic and corosolic acids, betulin, uvaol, and friedelin were obtained from Extrasynthese (Genay, France); and ursolic acid was purchased from Carl Roth (Karlsruhe, Germany). Helium used in GC-MS was purchased from Gazchema (Lithuania) and N,O-Bis(trimethylsilyl) trifluoro acetamide, Trimethylchlorosilane (BSTFA-TMCS) was acquired from Sigma-Aldrich.

Chemicals for anticancer activity assays—namely, 3-(4,5-dimethylthiazol-2-yl)-2,5-diphenyltetrazolium bromide (MTT) and crystal violet—were obtained from Sigma-Aldrich. Cell proliferation reagent WST-1 was from Roche Diagnostics (Basel, Switzerland). The plasticware for cell cultures was purchased from Corning (Corning, NY, USA) and Techno Plastic Products (Trasadingen, Switzerland). NanoShuttle magnetic nanoparticles were obtained from Nano3D Biosciences Inc. (Houston, TX, USA). Fetal bovine serum (FBS), Dulbecco’s modified Eagle high glucose medium (DMEM Glutamax), TrypLE^TM^ Express reagent, phosphate-buffered saline (PBS), and penicillin/streptomycin solution (100×) were acquired from Gibco (Thermo Fisher Scientific Inc., Waltham, MA, USA).

### 2.2. Cell Cultures

Human colon adenocarcinoma cell line (HT-29), human malignant melanoma cell line (IGR39), human clear renal carcinoma cell line (CaKi-1), and human endothelial cells (EC) CRL-1730 were obtained from the American Type Culture Collection (ATCC, Manassas, VA, USA). The cell culture medium consisted of DMEM Glutamax medium supplemented with 10% FBS and 1% antibiotics. Cells were kept at 37 °C with 5% CO_2_ in a humidified atmosphere and were grown to 70% confluence. During the passage, cells were trypsinized with 0.125% TrypLE™ Express solution and used till passage 20.

### 2.3. Plant Material

Lingonberry raw materials were collected in September 2019 from the wild at different natural sites in Lithuania (56°00′40.6″ N 25°31′29.4″ E, 55°59′20.6″ N 25°25′48.2″ E, 56°04′32.8″ N 25°29′41.1″ E, 55°35′45.1″ N 26°07′53.7″ E, 55°39′59.3″ N 25°59′26.7″ E (WGS)) and then pooled prior to extraction to obtain a homogeneous mixture of leaf and fruit samples, which were frozen (−20 °C) until analyses were performed. Lingonberry raw materials were identified according to morphological characteristics by Gabriele Vilkickyte and Lina Raudone. The voucher specimen was deposited in the herbarium of Vilnius University Siauliai Academy Botanical Garden.

### 2.4. Preparation of the Crude Extracts

To obtain lingonberry cuticular waxes, sample sets of entire leaves and fruits (10–20 g) were immersed in an adequate amount of chloroform to fully cover the sample (the sample/solvent approximate ratio of 1:8 was used for leaves and 1:2 for fruits) at room temperature and gently stirred for 30 s. As reported earlier [28,29,30], this short-term gentle extraction procedure precludes solvent penetration across the cuticle to deeper tissues and totally separates waxes from cutin, which is not soluble in chloroform. After dipping all samples, filtration through the glass fiber filter paper was performed and filtrates were combined into open containers which were placed in the well-ventilated chemical fume hood to evaporate. A further crystallization using methanol and re-evaporation to dryness was carried out, resulting in greenish or white solid fine powders—dry cuticular wax extracts of lingonberry leaves and fruits.

### 2.5. Fractionation Using Semi-Preparative HPLC

**Figure 1:**
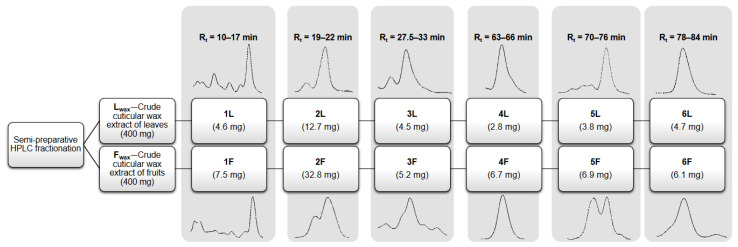
Scheme for the semi-preparative HPLC fractionation of the crude cuticular wax lingonberry extracts.

Fractionation of triterpenoids from crude cuticular wax extracts was conducted on a semi-preparative HPLC-PDA system (Waters, Milford, MA, USA) using an ACE C18 semi-preparative column (5 µm, 250 × 10 mm; ACT, Aberdeen, UK). The crude cuticular wax extracts were reconstituted with methanol and then filtered through a 0.45 µm membrane before injection into the system using an injection volume of 0.5 mL. Gradient elution at a constant flow rate (4 mL/min) was carried out with a total run time of 85 min as follows: starting from an isocratic run at 100% solvent A for 33 min, then increasing to 100% solvent B over 10 min and maintaining isocratic at 100% solvent B to the end of the run. Equilibration time was at least 15 min between runs. Solvent A was acetonitrile and water (89:11, v/v) and solvent B was methanol and acetonitrile (90:10, v/v). Fraction collection was based on time, monitoring the chromatograms at 205 nm and allowing the collection of known and unknown components to generate many different fractions with no more than two major peaks in each for the screening of anticancer agents. The fractionation scheme used in the present study is presented in Figure 1. Obtained fractions were evaporated to dryness and stored at 4 °C in air-tight containers until required.

### 2.6. Phytochemical Characterization Techniques

#### 2.6.1. HPLC-PDA for the Quantification of Triterpenoids and Other Compounds

Triterpenoid composition and purity of obtained fractions were analyzed using Waters e2695 Alliance HPLC system coupled with a PDA detector and validated method as reported previously [31]. Chromatographic separation of triterpenoids was carried out using ACE C18 column (3 µm, 150 × 4.6 mm; ACT, Aberdeen, UK). Before the 10 µL injection into the HPLC system, dry fractions were dissolved in methanol and filtered using 0.2 µm pore size membrane filters. The mobile phases and elution programs used were similar to those described in the semi-preparative HPLC method. For the analysis of triterpenoid acids (maslinic, corosolic, betulinic, oleanolic, and ursolic acids) and neutral triterpenoids with chromophores (fernenol, betulin, erythrodiol, and uvaol), isocratic elution consisting of acetonitrile and water (89:11, v/v) was used. The flow rate was 0.7 mL/min and the column temperature was maintained at 20 °C. Phytosterols (β-sitosterol, taraxasterol, and lanosterol) and neutral triterpenoids, which lack chromophores (lupeol, α-amyrin, β-amyrin, and friedelin) were determined using the mobile phase consisting of methanol and acetonitrile (90:10, v/v), eluted at isocratic mode at a flow rate of 1 mL/min when column temperature was set at 35 °C. The identification of all triterpenoids was made at a wavelength of 205 nm and quantification was performed using the standard dilution method according to 5–7 point linear calibration curves of external standards, except fernenol, which was tentatively quantified based on the calibration curve of betulin, as a substance with similar chemical structure.

All samples were additionally screened for the residuals of phenolic compounds using the same HPLC-PDA system and ACE Super C18 column (3 µm, 250 × 4.6 mm; ACT, Aberdeen, UK), exactly as described earlier [20]. A gradient elution system, comprised of 0.1% trifluoroacetic acid in water (eluent A) and acetonitrile (eluent B), was delivered at a flow rate of 0.5 mL/min when injection volume was 10 µL and the column temperature was 35 °C. Chromatographic peaks of phenolic compounds were identified by the retention time of the reference sample compared to the analyte, and the UV absorption spectra. For quantification, linear regression models were obtained using the standard dilution method.

#### 2.6.2. GC-MS for the Qualitative Analysis of Triterpenoids

To confirm the identity of triterpenoids in obtained fractions, additional GC-MS analysis was performed according to Caligiani et al. [32] and Sánchez Ávila et al. [33] with slight modifications. Prior to analysis, derivatization procedures were carried out. Methanolic solutions of cuticular wax extracts, obtained fractions, and reference compounds (100 µL) were evaporated to dryness under an N_2_ stream. Dried residues were reconstituted with 100 µL of DMF, silylated by adding 100 µL of BSTFA-TMCS (99:1), and held at 90 °C in a glycerol bath for 2 h. After derivatization, the resultant solutions were injected (1 μL; a split ratio was 1:10 (v/v)) with a Shimadzu autoinjector AOC-5000 (Shimadzu, Tokyo, Japan) for triterpenoid qualitative analysis using a robotic autosampler and a split/splitless injection port.

A Shimadzu GC/MS-QP2010nc Ultra chromatography system coupled to an Electron Ionization ion source and a single quadrupole MS (Shimadzu Technologies, Kyoto, Japan) and Rxi-5ms column (30 m, 0.25 mm × 0.25 µm; Restek, Bellefonte, PA, USA) were used. The oven temperature was programmed at 200 °C for 2 min increased to 300 °C at the rate of 4 °C/min and then increased to 320 °C at the rate of 10 °C/min and held for 6 min. The total run time was 35 min. Helium was used as the carrier gas at a flow rate of 1.07 mL/min. The detector ion source and interface temperatures were 200 °C and 280 °C, respectively. Mass spectra were acquired at an ionization voltage of 70 eV, a scan rate of 2500 *m*/*z*, and a scan time of 0.2 s., full-scan (*m*/*z* 29–700). Identification of triterpenoids was achieved by comparing their mass spectra with literature and library data from LabSolution GC/MS (version 5.71) (Shimadzu Corporation) and by comparison of their retention times and corresponding mass spectra with those of reference standards, where available.

### 2.7. Screening of Anticancer Agents

#### 2.7.1. MTT Assay

The effect of lingonberry cuticular wax extracts and obtained fractions on cancer and endothelial cell viability was determined using an MTT assay, according to Grigalius and Petrikaite [34]. HT-29, IGR39, CaKi-1, and EC lines were seeded in 96-well plates in triplicate in a volume of 100 µL (4000 cells/well) and pre-incubated at 37 °C for 24 h. Then, serial dilutions starting from 25 µg/µL of cuticular wax extracts and fractions (dissolved in DMSO) were made and added to the cells in triplicate, followed by incubation at 37 °C for 72 h. The medium in all the wells was replaced with the fresh one containing 0.5 mg/mL of MTT and subjected to further incubation for 4 h under the same conditions. The colored formazan crystals were dissolved in 100 μL of DMSO. Absorbance was measured at a test wavelength of 570 nm and a reference wavelength of 630 nm using a multi-detection microplate reader Multiskan GO (Thermo Fisher Scientific Oy, Ratastie, Finland). Medium without cells was used as a positive control and cells treated with 0.5% DMSO served as a negative control. EC_50_ values that represent the concentration, which is effective in reducing cell viability by 50%, were calculated using the Hill equation as described earlier [35].

$$y=E_{\max}+\left(\frac{E_{0}-E_{\max}}{1+{\left(\frac{D}{{\mathrm{EC}}_{50}}\right)}^{\mathrm{HS}}}\right)$$

where:

*y*—response measure at dose *D*;

*E*_0_ and *E*_max_—top and bottom asymptotes of the response;

EC_50_—concentration at half-maximal effect;

HS—slope parameter analogous to the Hill coefficient.

#### 2.7.2. Spheroid Growth and Viability Assay

Spheroids were formed using the magnetic 3D Bioprinting method as previously stated elsewhere [36]. Cancer cells were mixed with human fibroblast in a 6-well plate at the ratio of 1:1 and incubated at 37 °C in a humidified atmosphere containing 5% CO_2_ for 8 h. After magnetization, cells were trypsinized, centrifuged, and seeded into ultralow attachment 96-well plates in a volume of 100 µL (1500 cancer and 1500 human fibroblast cells/well). The magnetic drive (Nano 3D Biosciences, Inc., Houston, TX, USA) was used for the plate incubation at 37 °C in a humidified atmosphere containing 5% CO_2_ for 2 days to form spheroids. Then photos of spheroids were taken every 48 h using a phase-contrast Olympus IX73 inverted microscope (Olympus Corporation, Tokyo, Japan). The medium was replaced by the fresh one, containing 100% of solutions of the most promising samples (F_wax_, 1F, and 2F) at concentrations 5 and 25 μg/mL, every 48 h. ImageJ software (National Institutes of Health) was used to evaluate the effect of tested lingonberry samples on reducing the size of cancer spheroids, while the WST-1 assay was used to determine the change of cell viability in spheroids. Briefly, on the last day of the experiment, the medium of spheroids was replaced by the fresh one containing 10% of WST-1 and incubated for 6 h. Then 50 µL of solution from each well was transferred to the new 96-well plate and the absorbance was measured at 430 nm and a reference wavelength of 630 nm using a multi-detection microplate reader Multiskan GO. The spheroid viability was calculated using the following formula:

$$\mathrm{Relative}\;\mathrm{cell}\;\mathrm{viability}\;\left(\%\right)=\;\frac{{A\;-\;A}_{0}}{A_{\mathrm{NC}}{\;-\;A}_{0}}$$

where:

A—mean of absorbance of medium of spheroids treated with fractions;

A_0_—mean of absorbance of blank (no spheroids, only medium, positive control);

A_NC_—mean of absorbance of negative control (spheroids with no treatment).

### 2.8. Statistical Analysis

All data are presented as mean values (M) ± standard deviation (SD). The statistical significance of obtained results was established by one-way ANOVA with Tukey’s and Duncan’s post-hoc tests. The Pearson correlation method was used to evaluate correlations between data. IBM SPSS Statistics version 26.0 package, Microsoft Office Excel 2016, and GraphPad Prism 8 software were used for statistical analysis and visualization. Values of *p* < 0.05 were considered statistically significant.

## 3. Results and Discussion

### 3.1. Phytochemical Characterization

#### 3.1.1. Qualitative and Quantitative Analysis of Cuticular Wax Extracts

**Figure 2:**
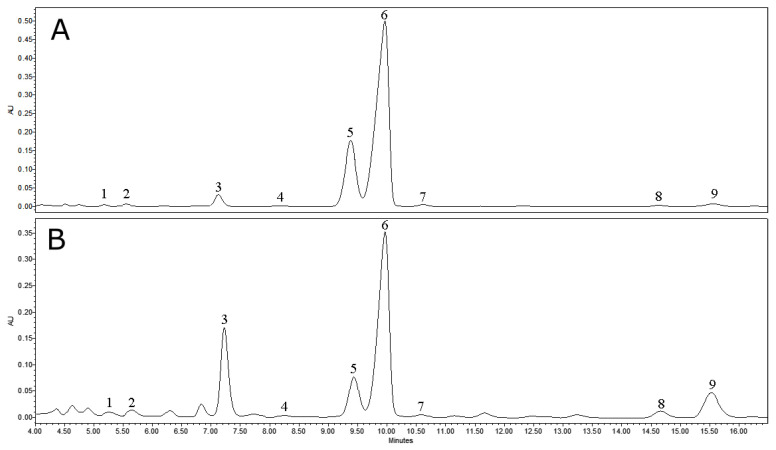
Representative HPLC-PDA chromatograms (λ = 205 nm) of triterpenoid acids and neutral triterpenoids with chromophores in cuticular wax extracts of lingonberry fruits (**A**) and leaves (**B**). Peak assignments: 1—maslinic acid; 2—corosolic acid; 3—fernenol; 4—betulinic acid; 5—oleanolic acid; 6—ursolic acid; 7—betulin; 8—erythrodiol; 9—uvaol.

**Figure 3:**
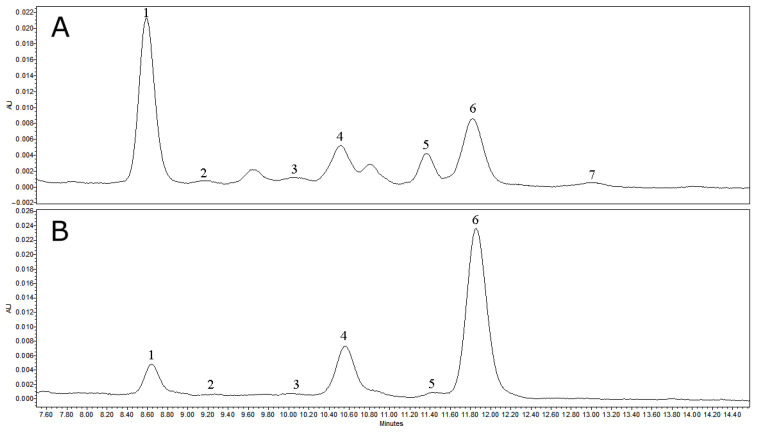
Representative HPLC-PDA chromatograms (λ = 205 nm) of phytosterols and neutral triterpenoids, which lack chromophores in cuticular wax extracts of lingonberry fruits (**A**) and leaves (**B**). Peak assignments: 1—lupeol; 2—lanosterol; 3—taraxasterol; 4—β-amyrin; 5—β-sitosterol; 6—α-amyrin; 7—friedelin.

Crude cuticular wax extracts of lingonberry leaves (L_wax_) and fruits (F_wax_) (Figure 2 and Figure 3) were evaluated for the qualitative and quantitative composition by the HPLC-PDA method as presented in Table 1 and Table 2. The complex of identified triterpenoids was comprised of five triterpenoid acids, eight neutral triterpenoids, and three phytosterols. The total amount of triterpenoids in cuticular wax extracts reached 291.01 mg/g DW (dry weight) in leaves and 824.05 mg/g DW in fruits. A similar level of triterpenoids in the lingonberry cuticle (553.9–730.5 mg/g wax extract), which was directly dependent on the phenological stages and highest in unripe fruits has previously been reported [37]. As stated by Jäger et al. [38], triterpenoids accumulate mainly in the surface cuticle or stem bark of plants. Significantly lower levels (41.5- and 61.5-fold) of total triterpenoids were found in methanolic extracts of entire lingonberry leaves and fruits, respectively [31], indicating that surface cuticle wax is a better source of triterpenoids than crude raw materials.

Triterpenoid acids were the major group determined in the cuticular wax extract of fruits accounting for 87.0% of the total identified triterpenoids. A significantly lower (5.3-fold) amount of triterpenoid acids was found in the cuticular wax extract of leaves. Ursolic acid prevailed in both samples as the principal compound, constituting 84.1% and 81.6% of identified triterpenoid acids in L_wax_ and F_wax_, respectively. These results were in line with the study of Dashbaldan et al. [37], wherein ursolic acid made up 79.1–82.4% of the complex of triterpenoid acids, determined in cuticular waxes of lingonberry fruits. However, this characteristic cannot be used alone for quality control of lingonberry, because other *Vaccinium* fruits, except bilberry, were also distinguished by the predominant isomer of ursolic acid [22,39,40]. Oleanolic acid was the second prevailing triterpenoid acid with a 7.0-fold higher content in F_wax_, compared to L_wax_. The complex of ursolic and oleanolic acid made up 86.5% of the total triterpenoids in the cuticular wax extract of fruits. A slightly lower contribution of these acids (70–73% to all triterpenoids) was found earlier in Finnish and Polish entire lingonberry fruits [41]. Other triterpenoid acids (betulinic, maslinic, and corosolic acids) were presented in relatively low amounts in L_wax_ and F_wax_ and were detected for the first time in cuticular waxes of lingonberry raw materials.

Neutral triterpenoids (fernenol, betulin, erythrodiol, uvaol, lupeol, β-amyrin, α-amyrin, and friedelin) comprised the second prevailing group of triterpenoids in F_wax_ and the major group in L_wax_, accounting for 11.8% and 53.0% of total identified triterpenoids, respectively. This is consistent with previous results [31], wherein the richness of neutral triterpenoids in entire lingonberry leaves was reported. The principal neutral triterpenoid of L_wax_ was α-amyrin (47.4% of neutral triterpenoids), followed by fernenol and uvaol, while F_wax_ was characterized by the abundance of lupeol (32.7% of neutral triterpenoids). As found by other researchers [37,41] main neutral triterpenoids in entire lingonberry raw materials and waxes of fruits were fernenol, lupeol, and α-amyrin. It was noted that the content of neutral triterpenoids is highly dependent on the age of raw materials and geographical or seasonal factors since they play an important role in plant defense. Previous compositional analysis of the cuticular wax of lingonberry fruits [22] indicated that the content of α- and β-amyrins can even surpass the content of triterpenoid acids. Minor neutral triterpenoid, friedelin, was found only in F_wax_ (4.8% of neutral triterpenoids), while L_wax_ did not contain this compound. This is distinguishable from extracts from entire leaves, in which 1.0–6.9% friedelin contribution to neutral triterpenoids was reported [31,41].

The content of phytosterols in cuticular waxes of lingonberry fruits and leaves was very low, only 0.4–1.2% of the total triterpenoids. Previous data [31] indicate that the contribution of phytosterols to total triterpenoids (6.8–7.4%) in entire lingonberry raw materials is considerably higher than in the case of cuticular waxes. The content of phytosterols in cuticular waxes of lingonberry may increase up to 45% during ripening [37]. Our determined complex of phytosterols was included of β-sitosterol and traces of taraxasterol and lanosterol. In agreement with Vrancheva et al. [42], β-sitosterol can be regarded as the prevailing phytosterol of lingonberry raw materials. The content of this compound was 7.1-fold higher in the cuticular wax of fruits than that of leaves.

Results of GC-MS analysis (Table 3) confirmed the identity of all previously mentioned triterpenoids and proposed the presence of eight other unidentified minor compounds in cuticular waxes of lingonberry raw materials. Based on the triterpenoid GC-MS elution order and known fragmentation pathways, a tentative assignment to the triterpenoid group was suggested. Tentative assignment of unidentified compounds to phytosterols I–III was based on similar *m*/*z* values as reported for plant sterols earlier [39]. Furthermore, these compounds shared fragment ion *m*/*z* at 129, which is one of the base peaks of phytosterols, having a double bond on the B ring [43]. Exact identity could not be determined because of similar mass spectra and abundance of different phytosterols in lingonberry raw materials—namely, campesterol, stigmasterol, stigmastanol, cycloartenol, cycloartenyl acetate, and 24-methylenecycloartanol, as reported previously [37,42]. This may constitute the object of future studies. Phytosterols II and III were determined in both extracts of cuticular wax extracts, while phytosterol I was found only in leaf samples. Unidentified compounds were tentatively assigned to unknown neutral triterpenoids I and II because of fragment ions at *m*/*z* 218 and 189, which are abundant in pentacyclic triterpene alcohols [43]. In the light of prior reports [22,41] it is conceivable that lingonberry raw materials can contain the following neutral triterpenoids: adriaticol, swertenol, and ursenal. Unknown neutral triterpenoid I was found in cuticular waxes of both raw materials, while neutral triterpenoid II was found only in waxes of fruits. Researchers [26,42,44] indicated that lingonberry raw materials can also contain esterified, acetylated, and methylated triterpenoid acids or aldehydes, such as oleanolic and ursolic aldehydes and acetates. The tentative identity of these compounds could be suggested to unknown derivatives of triterpenoid acids I–III, which shared the same fragment ion *m*/*z* at 320, indicating the presence of ester group in C-17 [45] and *m*/*z* at 203, which is a typical mass ion fragment of oleanane and ursane skeletons [46]. Derivatives of triterpenoid acids I and II were detected in L_wax_ and F_wax_, while the derivative of triterpenoid acids III was determined only in F_wax_.

The authors of [22,47] suggested that non-purified lingonberry cuticular extracts are supplemented by other than triterpenic compounds, namely di-ketones, sterols, isoprenoids, alkanes, alkenes, aldehydes, carbohydrates, alcohols, fatty acids, and phenolic acids. Our results indicate that level of residual compounds is 4-fold higher in L_wax_ than that in F_wax_. Chlorophyll also could be one of the main residuals in L_wax_ since it cannot be removed during the extraction with chloroform [48]. Additional screening for phenolic compounds in cuticular waxes confirmed the presence of phenolic acids. F_wax_ contained a considerable amount of phenolic acids precursors—benzoic and *trans*-cinnamic acids (71.27 ± 10.79 and 2.81 ± 0.25 mg/g DW), while 86-times lower content of these acids was found in L_wax_. Furthermore, trace levels of *p*-coumaric, sinapic, and ferulic acids were detected in both cuticular wax extracts. Klavins et al. [47] reported an 8.1-fold higher level of benzoic acid in the cuticular wax of lingonberry fruits than our determined. These differences can be explained in part by the short-term gentle extraction procedure used in our study, which preserved the complete dissolution of intermediate polarity compounds, such as phenolic acids in the extraction solvent.

#### 3.1.2. Qualitative and Quantitative Analysis of Obtained Fractions

First fractions from cuticular wax extracts of lingonberry leaves and fruits (1L and 1F), collected at the beginning of semi-preparative HPLC analysis, were pure from phenolic acids residuals, which were eluted with void volume due to higher polarity. They contained the following triterpenoids: three triterpenoid acids (maslinic, corosolic, and betulinic acids) and one neutral triterpenoid (fernenol) (Table 1 and Table 2). Furthermore, three unidentified compounds (unknown triterpenoid acids I and II and unknown neutral triterpenoid I) were tentatively determined in these samples (Table 3). A slightly (1.4-fold) higher content of triterpenoid acids was found in 1F when compared to 1L. The prevailing component of 1L and 1F was fernenol, which comprised 80.5% and 67.9% of these fractions, respectively. Since fernenol, isoarbinol, and their derivatives are rarely mentioned as wax constituents, particularly abundant in vascular plants such as ferns [49], the richness of fernenol makes lingonberry raw materials an easily accessible alternative source for the purification of this compound.

Fractions 2L and 2F were characterized by the complex of oleanolic and ursolic acids. It is extremely difficult to separate these structural isomers from each other due to very similar chemical properties. Therefore, the pharmacological effects of the combination of oleanolic and ursolic acids are usually studied and commonly used in clinical practices [50,51]. Low content (1.5–2.1%) of betulin as an impurity was found in 2L and 2F. Ursolic acid was the prevailing compound in both samples, accounting for 77.0% and 67.3% of 2L and 2F respectively. The content ratio of oleanolic:ursolic acids was found to be 1:3.7 in a fraction of leaves and 1:2.2 in a fraction of fruits. Previous findings [31,37,41,52] indicated that the ratio of oleanolic:ursolic acids could vary significantly from 1:1.2–5.8 in leaves and 1:1.8–1:5.3 in fruits depending on geographical origins and other abiotic factors.

Third fractions of cuticular wax extracts were enriched by neutral triterpenoid uvaol, which made up 87.0% and 80.1% of 3L and 3F, accordingly. The second prevailing compound was erythrodiol, which has a similar chemical structure and molecular weight to uvaol, thus they are usually analyzed together [53]. Ratios of these triterpenic dialcohols (erythrodiol:uvaol) were 1:6.7 in 3L and 1:4.0 in 3F. Our experiments corroborate the study of Trivedi et al. [22] and suggest that cuticular waxes of lingonberries could be the potential source of uvaol. This characteristic may be highlighted as specific since cuticular waxes of other *Vaccinium* plants did not distinguish by the abundance of uvaol [39,54]. The 3L fraction was found to be purer as unknown neutral triterpenoid II and derivative of triterpenoid acid III were additionally detected in 3F.

Fractions 4L and 4F contained one principal neutral triterpenoid—lupeol. Fraction 4F was pure and no other triterpenoids were detected, while 4L was characterized by the presence of impurities—lanosterol (1.5% to identified triterpenoids) and one unidentified compound (unknown phytosterol I). Results indicate that due to differences in lingonberry matrixes, cuticular waxes of lingonberry fruits seem to be a better source for the isolation of lupeol. Furthermore, the richness of this compound in lingonberry fruits could be noted. The content of lupeol in the cuticular wax of lingonberry fruits was the highest among tested Northern berries [22]. Recently lupeol was isolated by different chromatographic methods from stem barks of *Parkia biglobosa* (Jacq.) G. Don [55], *Zanthoxylum monogynum* A.St.-Hil. [56], and root extract of *Grewia flava* DC. [57].

Fifth fractions of cuticular wax extracts of leaves and fruits differed from each other by predominant compounds. Fraction 5L was distinguished by the high content of β-amyrin, which comprised 87.3% of identified compounds, and significantly lower levels of the following sitosterols: β-sitosterol (9.5%), taraxasterol (2.7%), and lanosterol (0.6%). Whereas the main component of 5F was β-sitosterol, accounting for 52.1% of identified compounds; followed by β-amyrin (42.5%), taraxasterol (3.7%), and lanosterol (1.7%). The sum amount of phytosterols was 4.2 times higher in the fifth fraction of fruits than that of leaves. Two more unidentified compounds (unknown phytosterol II and III) were found in samples 5L and 5F. The complex of β-sitosterol and lanosterol from cuticular waxes of lingonberry fruits was fractionated previously and obtained ratio of these phytosterols was found to be 1:1.1 [47].

The last fraction of cuticular waxes of leaves (6L) can be regarded as a pure fraction of α-amyrin as no more compounds were identified. α-Amyrin was earlier highlighted as a phytochemical marker of lingonberry leaves, which highest content was found in old lingonberry leaves (collected from the bottom of the stem) with an increasing tendency during colder months [52]. The last fraction of cuticular waxes of fruits (6L) was comprised of α-amyrin as the main component (76.9% of identified triterpenoids), followed by friedelin (13.9%) and β-sitosterol (9.2%).

### 3.2. Anticancer Activity of Cuticular Wax Extracts and Obtained Fractions

#### 3.2.1. Cytotoxicity Study in 2D Cultures

**Figure 4:**
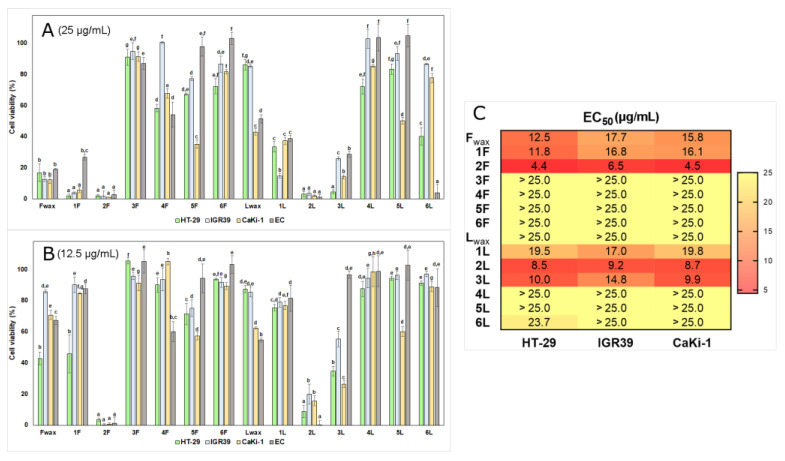
Cytotoxicity of cuticular waxes (L_wax_, F_wax_) and fractions (1–6L and 1–6F) of lingonberry leaves and fruits in 2D cell cultures (cell monolayers). (**A**) Cell viability in HT-29, IGR39, CaKi-1, and EC lines at a concentration of 25 µg/mL. (**B**) Cell viability in HT-29, IGR39, CaKi-1, and EC lines at a concentration of 12.5 µg/mL. (**C**) EC_50_ values against cancer cell lines. Bars marked with different letters indicate statistically significant differences (*p* < 0.05) within the same category.

Cuticular wax extracts of lingonberry leaves and fruits and obtained fractions were found to reduce the viability of human cancer cells at concentrations of 12.5 and 25.0 µg/µL (Figure 4). The highest concentration was chosen based on the solubility of triterpenoids and previous studies. Since exact EC_50_ values could not be determined for all tested fractions, Table S1, summarizing phytochemical features and average cytotoxicity based on both concentration levels, was additionally provided. Strong positive correlations (*r* = 0.805–0.905, *p* < 0.05) towards different cancer cells were determined. In general, higher cytotoxicity was determined towards HT-29 and CaKi-1 cell lines than against IGR-39 cells. Cytotoxicity against endothelial cells was also correlated with cytotoxicity against cancer cells (*r* = 0.642–0.832, *p* < 0.05) with the lowest correlation to the CaKi-1 cell line. Tissue selectivity is a major issue of conventional chemotherapeutic antitumor drugs [58]. Agents that are more effective in tumor tissues than in normal counterpart cells can be considered for the potential development of selective anti-cancer drugs [59]. Cytotoxicity to normal cells, as well as to cancer cells, was concentration-dependent (*r* = 0.661–0.882, *p* < 0.05).

At a concentration of 25 µg/µL, crude cuticular wax extract of fruits (F_wax_) was 3.5–5.1 times more active against different cancer cells than that of leaves (L_wax_). The observed difference might be explained by the significantly higher contribution of triterpenoids to the wax extract of fruits as discussed previously. F_wax_ showed higher selectivity than L_wax_ with up to 1.6-fold higher cytotoxicity to cancer cells than for normal endothelial cells. The obtained EC_50_ values towards cancer cells were in a range of 12.5–17.7 µg/mL for F_wax_, and >25.0 µg/µL for L_wax_. Cuticular wax extracts enriched with triterpenoids were found to be considerably more active against the same cancer cells than crude extracts of entire lingonberry raw materials enriched with phenolic compounds as determined earlier. Previously obtained EC_50_ values were in a range of 1100–1500 µg/mL for the crude extract of entire lingonberry fruits and 120–170 µg/mL for the leaves [20]. Besides anticancer activity, cuticular wax extract and fractions of lingonberry fruits have been shown to have potential UV-B blocking and antimicrobial activity [47].

Fraction 1F was among the most active fractions and at a concentration of 25 µg/µL reduced cancer cells viability by 94.5–98.1%. Furthermore, this fraction was distinguished by the highest selectivity—observed cytotoxicity to normal cells was 4.9–14.4 times lower than for cancer cells. Counterpart fraction 1L at a concentration of 25 µg/µL was about 10-fold less active against cancer cell lines with the greatest differences towards HT-29 cell line. Maslinic, corosolic, and betulinic acids have been reported to be promising compounds for the chemoprevention or treatment of colon cancer by inhibiting proliferation and inducing apoptosis in HT-29 and other colon cancer cell lines [60,61,62], thus suggesting that observed higher activity of 1F was in part of the higher contribution of these acids. Melanoma is considered an intrinsically drug-resistant tumor, which is extremely aggressive and prone to metastasis [63]. Surprisingly, fraction 1L at a concentration of 12.5 µg/µL significantly surpassed 1F by cytotoxic activity towards human malignant melanoma IGR-39 cell lines, resulting in similar EC_50_ values (16.8–17.0 µg/µL). The higher contribution of the predominant compound fernenol in 1L when compared to 1F implies the potency of this compound in targeted therapy of melanoma. Previously, fernenol showed the highest fungicidal activity among several tested triterpenoids [64] and significant selective cytotoxicity against human acute monocytic leukemia cell line (THP-1) [65].

There was no significant difference between cytotoxicity at a concentration of 25 µg/µL against cancer cell lines between 1F and fractions 2F and 2L, which were enriched with oleanolic and ursolic acids. Slightly higher activity at a lower tested concentration (12.5 µg/µL) and up to 2-times lower EC_50_ values were calculated for 2F (EC_50_ = 4.4–6.5 µg/mL), which distinguished from 2L by a lower ratio of oleanolic:ursolic acids (lower contribution of ursolic acid). Ursolic and oleanolic acids are among the most commonly investigated triterpenoids with well-documented anticancer properties. However, most studies have focused on the activity displayed with either oleanolic or ursolic acids alone. According to the literature reviewed [66,67,68,69], half maximal effective or inhibitory concentrations of these acids individually towards human leukemia, breast, gastric, cervical, and hepatocellular carcinoma cell lines were in a range of 6.4–72.0 µg/mL. It was noted that different position of one methyl group in the chemical structure of these acids affects the stability and affinity towards reactants and thus play a crucial role in antioxidant and anticancer activities [70]. Authors [71,72] disclosed that the pharmacological interaction of oleanolic and ursolic acids which usually coexist naturally in plants as a complex may produce an ideal effect and reported that a mixture of these compounds possessed stronger biological effects than separately. As the synergistic activity of oleanolic and ursolic acids was highlighted [51], it is particularly important to study combined effects in different proportions of these compounds.

Counterpart fractions 3F and 3L showed different activity against cancer cells with significantly stronger activities of the fraction of leaves at both concentration levels. This seems to be dependent on the higher contribution of uvaol and purity of erythrodiol:uvaol complex. The greatest difference in cytotoxicity was observed against the HT-29 cell line at the concentration level of 25 µg/mL—the activity of 3L was up to 20.1-fold higher compared to 3F. The observed EC_50_ of 3L ranged from 9.9 µg/mL towards CaKi-1 up to 14.8 µg/mL against the IGR39 cell line. Moreover, this uvaol-rich fraction was characterized by relatively good selectivity to HT-29 and CaKi-1 cancer cell lines. Cytotoxicity values towards these cancer cell lines were 2.8–6.3 and 2.0–3.5 times higher than for normal EC at different concentration levels. Results of Bonel-Pérez et al. [73] showed that uvaol has a clear and selective anticancer activity in human hepatocellular carcinoma HepG2 cell line (IC_50_ = 14.1 µg/mL after 72 h of incubation) supported by strong anti-migratory capacity. Similar profound activity towards the HepG2 cell line was observed for erythrodiol (IC_50_ = 8.3 µg/mL after 72 h of incubation) [74], which was the second principal component of 3F and 3L fractions. Furthermore, erythrodiol and uvaol were found to have potential in the prevention and treatment of brain tumors [75]. It was previously suggested that bioactivities of erythrodiol and uvaol could be even higher than for triterpenoid acids due to free two hydroxyl groups in remote positions [76].

Fractions, which were enriched with lupeol (4F and 4L) were significantly more active against HT-29 and CaKi-1 cell lines compared to IGR-39. However, higher than 15% (14.6–41.6%) cytotoxicity towards cancer cells was only observed at a concentration of 25 µg/mL, and cytotoxicity of 4F and 4L was significantly weaker when compared to F_wax_, L_wax_, 1F, 1L, 2F, 2L, and 3L. Fraction 4F showed slightly (up to 1.3 times) higher activity than 4L, which was characterized by impurities of phytosterols. The EC_50_ values of 4F and 4L were reported to be greater than 25.0 µg/mL (>58.6 µM for 4F as for pure fraction of lupeol). Several researchers suggested that lupeol has stronger cytotoxicity towards other than our tested cancer cell lines, namely human glioblastoma U87MG.ΔEGFR (EC_50_ = 13.6 µM), colon colorectal cancer HCT116 (IC_50_ = 19.6 µM) [77], T-lymphoblastic leukemia (IC_50_ = 27.6 µM), multiple myeloma RPMI 8226 (IC_50_ = 37.5 µM) [78] cell lines, and defined lupeol as very promising new generation multitarget drug, which can induce cancer cell apoptosis and inhibit proliferation, cell migration, and invasion [79].

Significantly lower cytotoxicity was found for 5L compared to 5F, which was distinguished by the higher contribution of phytosterols. The strongest cytotoxicity was observed towards renal CaKi-1 cell line—5L and 5F reduced viability of these cells by 49.8–64.8% at a concentration of 25 µg/mL. At the lower tested concentration (12.5 µg/mL) cytotoxicity against this cell line was even higher (*p* < 0.05) than found of F_wax_, L_wax_ 1F, and 1L. Moreover, 5L and 5F fractions were approximately twice more active towards the CaKi-1 cell line than the normal EC. It has previously been concluded that β-sitosterol, the principal phytosterol of 5L and 5L, has a strong potential for suppressing neoplastic transformation in experimental renal cancer and can be used in a variety of diseases without significant toxicity [80,81]. Mounting evidence supports the protective role of phytosterols from various types of cancer through inhibition of carcinogen production, cancer cell growth, invasions, angiogenesis, and induction of cell cycle arrest, as well as reducing oxidative stress [82].

A pure fraction of α-amyrin from the cuticular wax of lingonberry leaves (6L) was characterized by the highest activity towards the HT-29 cell line. At a concentration of 25 µg/mL, the cytotoxicity of 6L towards the HT-29 cell line was 1.8-fold higher than that of 6F, which besides α-amyrin, contained residuals of β-sitosterol and friedelin and 2.1-fold greater than that of 5L, which in addition to phytosterols contained β-amyrin as prevailing triterpenoid. These results support the superiority of α-amyrin compared to β-amyrin or phytosterols in terms of cytotoxicity against colon cancer HT-29 cells. Nevertheless, a concentration of 25 µg/mL seems to be not selective against cancer cells when compared to EC and further cytotoxicity studies with more specific normal colon fibroblast are needed. The obtained EC_50_ = 23.7 µg/mL (or 55.5 µM as for pure α-amyrin fraction) of 6L towards HT-29 was compatible with EC_50_ = 19.5 µg/mL of L_wax_, which also contained α-amyrin as prevailing neutral triterpenoid. Gonçalves Pereira et al. [83] reported that α-amyrin possessed higher and more selective cytotoxicity against THP-1 and K562 leukemia cell lines (IC_50_ = 9.9 µM and 8.5 µM, respectively) than that of β-amyrin, β-sitosterol, and friedelin derivatives. Another study [84] provided further evidence for α-amyrin potential towards colon cancer and outlined selective cytotoxicity of sub-fraction from *Mesua ferrea* L. stem bark, which was enriched with α-amyrin (IC_50_ = 6.7 µM and 13.1 µM for colon cancer cell lines HCT 116 and HT-29, respectively).

#### 3.2.2. Effect on 3D Cultures (Spheroids) Growth and Viability

**Figure 5:**
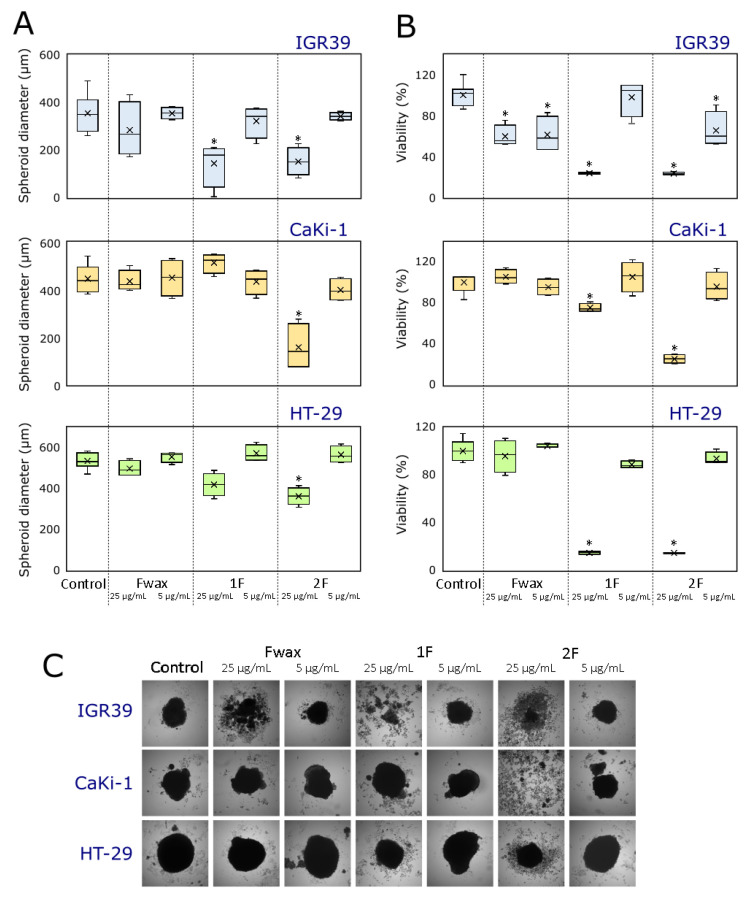
Cytotoxicity of F_wax_, 1F, and 2F in 3D cell cultures. (**A**) Diameter of HT-29, IGR39, and CaKi-1 spheroids at concentrations of 5 and 25 µg/mL. (**B**) Cell viability in HT-29, IGR39, and CaKi-1 spheroids at concentrations of 5 and 25 µg/mL. (**C**) Photos of cancer spheroids after 8 days. Asterisks (*) indicate statistically significant differences (*p* < 0.05) compared to the control (untreated spheroids).

The effects of the most promising samples (F_wax_, 1F, and 2F) on HT-29, IGR39, and CaKi-1 cancer spheroid growth at concentrations of 5 and 25 µg/mL are depicted in Figure 5. Although 2D monolayer models are the most commonly used type of cancer cells due to simplicity and low-cost maintenance, they have limitations regarding different cell interactions and lack of cell structural architecture. To overcome these problems, three-dimensional cell culture models emerged as a novel promising, and more reliable tool for the development of new drugs, exhibiting more similar behavior and microenvironment of cells to in vivo system [85,86]. Nevertheless, the viability of different 2D cancer cell lines was well correlated with the viability of the same 3D cultures at a concentration of 25 µg/mL of tested fractions (*r* = 0.921–0.984, *p* < 0.05), indicating that our results obtained in the 2D model are also reliable for initial screening.

This study showed that spheroid diameter positively correlated with spheroid viability (*r* = 0.635–0.830), however, correlations were not significant (*p* > 0.05). The authors in [87] indicated that the total spheroid size is not directly proportional to the number of living cells because of different cell density, intracellular spaces, or central necrosis during growth, therefore cell viability also should be taken into consideration when analyzing results. Although cuticular wax extract of fruits (F_wax_) had no significant effect on the size of spheroids, a significant reduction (about 1.7-fold, compared to control) was observed in the viability of IGR-39 3D culture at both tested concentrations (5 and 25 µg/mL). In general, F_wax_ was distinguished by the lower activity when compared to tested fractions (1F and 2F), with respective 1.2-fold and 1.5-fold weaker inhibition of cancer spheroids growth and viability on average. This suggests that particular triterpenoid mixtures from lingonberry waxes are more potent as anti-cancer agents than crude unpurified extract.

Fraction 1F, which was enriched with fernenol, maslinic, corosolic, and betulinic acids significantly reduced the viability of all spheroids (HT-29 by 84.7%, IGR-39 by 75.1%, CaKi-1 by 24.5% compared to control) and the actual IGR-39 spheroid size (by 58.1%) at a concentration of 25 µg/mL. These findings seem to support our previous statements about the strong cytotoxic activity of 1F and its possible application in the treatment of colon and renal cancer. Butkeviciute et al. [88] noted that corosolic acid alone had the strongest capacity to reduce cancer spheroid growth and initiate spheroid disintegration compared with betulinic, ursolic, and oleanolic acids, thus suggesting that corosolic acid might be one of the biological activity markers responsible for strong anticancer properties of 1F. Due to strong anticancer potency, future research should consider further purification and structure elucidation of unidentified compounds in 1F, followed by pharmacological characterization as well as a structure–activity relationship investigation.

Similar to 1F, profound anticancer activity was observed in fraction 2F, namely a mixture of oleanolic:ursolic acids (1:2.2) obtained from the cuticular wax of fruits. At a concentration of 25 g/mL, this fraction reduced cell viability by 73.7–85.2% and inhibited spheroids growth by 31.4–62.9%. Moreover, the clear disintegration of spheroids affected by 2F could be seen at the end of the experiment. A significant reduction in viability (by 33.9%) of IGR39 spheroids was found at a lower tested concentration (5 g/mL), which was comparable with the EC_50_ values of this fraction as determined previously (4.4–6.5 g/mL). Piet and Paduch [89] reported that ursolic and oleanolic acids, administered in combination, significantly inhibited viability and motility of colorectal cancer cells through uPA/uPAR downregulation and modulation of cellular E-cadherin levels, and exhibited protective properties towards normal cells. Another recent research [90] implied that apple peel extracts enriched with major triterpenoids—ursolic and oleanolic acids more effectively reduced the viability of HT-29 spheroids than individual ursolic or oleanolic acids when supplemented with doxorubicin. Taken together, it can be concluded that combinations of ursolic and oleanolic acids increase their anticancer potential. Therefore, the complex of these acids obtained from cuticular waxes of lingonberry fruits can be considered a promising therapeutic candidate, with potential benefits in the management of cancer diseases.

## 4. Conclusions

This study first fractionated triterpenoids from cuticular waxes of lingonberries to identify anticancer agents. Chromatographic analyses (HPLC-PDA and GC-MS) indicated the presence of five triterpenoid acids, eight neutral triterpenoids, three phytosterols, and eight minor unidentified triterpenoids in cuticular waxes of lingonberry leaves or fruits. As a result of semi-preparative HPLC fractionation, 12 fractions enriched with particular triterpenoids or their mixtures were obtained in total. Cytotoxicity studies carried out by MTT assay towards HT-29, IGR39, and CaKi-1 cancer cells, as well as normal endothelial cells, highlighted the most efficient fractions and their predominant compounds, with EC_50_ values ranging from 4.4 to >25 µg/mL. Cuticular wax extract of lingonberry fruits (F_wax_) and fractions—containing maslinic, corosolic, and betulinic acids plus fernenol (1F) and complex of oleanolic:ursolic acids (2F)—were further evaluated for cytotoxicity in 3D cancer cell cultures, showing the ability of these samples to initiate disintegration of cancer spheroids and reduce their viability and growth. Our findings support the use of lingonberry cuticular waxes as a source for the isolation of particular triterpenoids or their complexes, which could be used in the development of new therapeutic candidates for the management of colon, melanoma, or renal cancer diseases.

## Figures and Tables

**Table 1:** Content of triterpenoids (mg/g DW ± SD) in cuticular waxes (L_wax_) and fractions (1–6L) of lingonberry leaves, determined by HPLC-PDA methods.

Triterpenoid	L_wax_	1L	2L	3L	4L	5L	6L
Maslinic acid	0.59 ± 0.04 ^h,i^	39.94 ± 1.86 ^c^	ND	ND	ND	ND	ND
Corosolic acid	2.74 ± 0.12 ^g,i^	88.53 ± 3.90 ^b^	ND	ND	ND	ND	ND
Betulinic acid	0.14 ± 0.01 ^h^	4.17 ± 0.12 ^d^	ND	ND	ND	ND	ND
Oleanolic acid	18.12 ± 0.60 ^d^	ND	195.77 ± 4.74 ^b^	ND	ND	ND	ND
Ursolic acid	113.79 ± 2.00 ^a^	ND	718.99 ± 27.51 ^a^	ND	ND	ND	ND
Sum of triterpenoid acids	135.38	132.64	914.76	-	-	-	-
Fernenol	29.40 ± 1.08 ^c^	549.24 ± 19.26 ^a^	ND	ND	ND	ND	ND
Betulin	2.56 ± 0.08 ^g,i^	ND	19.38 ± 0.69 ^c^	ND	ND	ND	ND
Erythrodiol	3.46 ± 0.09 ^g^	ND	ND	130.99 ± 6.32 ^b^	ND	ND	ND
Uvaol	23.31 ± 0.83 ^c^	ND	ND	874.47 ± 39.46 ^a^	ND	ND	ND
Lupeol	7.42 ± 0.21 ^f^	ND	ND	ND	848.47 ± 35.14 ^a^	ND	ND
β-Amyrin	15.11 ± 0.55 ^e^	ND	ND	ND	ND	762.69 ± 22.31 ^a^	ND
α-Amyrin	73.11 ± 1.38 ^b^	ND	ND	ND	ND	ND	994.24 ± 18.96 ^a^
Friedelin	ND	ND	ND	ND	ND	ND	ND
Sum of neutral triterpenoids	154.37	549.24	19.38	1005.47	848.47	762.69	994.24
Lanosterol	NQ	ND	ND	ND	12.53 ± 0.37 ^b^	5.15 ± 0.18 ^c^	ND
Taraxasterol	NQ	ND	ND	ND	ND	23.45 ± 0.77 ^c^	ND
β-Sitosterol	1.29 ± 0.04 ^g,h^	ND	ND	ND	ND	82.62 ± 2.44 ^b^	ND
Sum of phytosterols	1.29	-	-	-	12.53	111.22	-
Total identified	291.01	681.88	934.14	1005.47	861.00	873.91	994.24

Different letters represent statistically significant differences (*p* < 0.05) between contents of triterpenoids within the same sample; ND—not detected, NQ—not quantified.

**Table 2:** Content of triterpenoids (mg/g DW ± SD) in cuticular waxes (F_wax_) and fractions (1–6F) of lingonberry fruits, determined by HPLC-PDA methods.

Triterpenoid	F_wax_	1F	2F	3F	4F	5F	6F
Maslinic acid	1.09 ± 0.04 ^f^	66.09 ± 2.46 ^c^	ND	ND	ND	ND	ND
Corosolic acid	3.22 ± 0.13 ^e,f^	105.20 ± 4.08 ^b^	ND	ND	ND	ND	ND
Betulinic acid	0.31 ± 0.01 ^f^	15.74 ± 0.48 ^d^	ND	ND	ND	ND	ND
Oleanolic acid	127.57 ± 4.86 ^b^	ND	335.65 ± 13.63 ^b^	ND	ND	ND	ND
Ursolic acid	584.96 ± 21.42 ^a^	ND	723.73 ± 33.64 ^a^	ND	ND	ND	ND
Sum of triterpenoid acids	717.15	187.03	1059.38	-	-	-	-
Fernenol	17.05 ± 0.68 ^d^	395.41 ± 7.78 ^a^	ND	ND	ND	ND	ND
Betulin	3.54 ± 0.14 ^e,f^	ND	15.82 ± 0.68 ^c^	ND	ND	ND	ND
Erythrodiol	2.07 ± 0.08 ^e,f^	ND	ND	133.88 ± 2.38 ^b^	ND	ND	ND
Uvaol	10.78 ± 0.34 ^d,e^	ND	ND	539.29 ± 14.73 ^a^	ND	ND	ND
Lupeol	31.71 ± 1.46 ^c^	ND	ND	ND	1004.90 ± 46.23 ^a^	ND	ND
β-Amyrin	10.86 ± 0.22 ^d,e^	ND	ND	ND	ND	348.80 ± 13.36 ^b^	ND
α-Amyrin	16.38 ± 0.49 ^d^	ND	ND	ND	ND	ND	663.80 ± 14.28 ^a^
Friedelin	4.70 ± 0.18 ^e,f^	ND	ND	ND	ND	ND	119.65 ± 3.65 ^b^
Sum of neutral triterpenoids	97.09	395.41	15.82	673.17	1004.90	348.80	783.45
Lanosterol	0.10 ± 0.00 ^f^	ND	ND	ND	ND	13.82 ± 0.42 ^c^	ND
Taraxasterol	0.57 ± 0.03 ^f^	ND	ND	ND	ND	30.65 ± 0.85 ^c^	ND
β-Sitosterol	9.14 ± 0.29 ^d,e^	ND	ND	ND	ND	427.79 ± 15.51 ^a^	79.36 ± 2.75 ^c^
Sum of phytosterols	9.81	-	-	-	-	472.26	79.36
Total identified	824.05	582.44	1075.20	673.17	1004.90	821.06	862.81

Different letters represent statistically significant differences (*p* < 0.05) between contents of triterpenoids within the same sample; ND—not detected.

**Table 3:** GC-MS data of triterpenoids identified in cuticular waxes (L_wax_, F_wax_) and fractions (1–6L and 1–6F) of lingonberry leaves and fruits.

Triterpenoid	Retention Time, min	Main Fragment Ions, *m*/*z* (Relative Intensity %)	Where Identified
Unknown phytosterol I	23.82	386 (3), 329 (15), 129 (30), 105 (28), 95 (42), 73 (95), 69 (47), 57 (35), 43 (35), 41 (45)	L_wax_, 4L
Lanosterol	24.94	426 (3), 393 (20), 129 (25), 109 (65), 95 (72), 73 (70), 69 (100), 55 (41), 41 (92)	L_wax_, F_wax_, 4L, 5L, 5F
β-Sitosterol	25.01	414 (8), 396 (15), 357 (14), 145 (15), 129 (68), 95 (23), 73 (62), 55 (35), 57 (56), 43 (100), 41 (29)	L_wax_, F_wax_, 5L, 5F, 6F
β-Amyrin	25.23	426 (4), 218 (100), 203 (33), 189 (24), 107 (32), 95 (60), 73 (90), 69 (54), 55 (29)	L_wax_, F_wax_, 5L, 5F
Unknown phytosterol II	25.56	410 (4), 374 (16), 269 (17), 174 (20), 129 (33), 121 (45), 107 (26), 73 (100), 69 (61), 43 (2), 41 (32)	L_wax_, F_wax_, 5L, 5F
α-Amyrin	25.75	426 (7), 218 (100), 203 (20), 189 (31), 107 (32), 95 (43), 73 (64), 55 (47)	L_wax_, F_wax_, 6L, 6F
Unknown neutral triterpenoid I	25.79	426 (3), 218 (49), 201 (51), 189 (25), 121 (34), 109 (62), 107 (57), 95 (47), 73 (100), 69 (90), 55 (50)	L_wax_, F_wax_, 1L, 1F
Lupeol	25.89	426 (3), 218 (13), 189 (24), 129 (38), 109 (37), 95 (42), 73 (100), 68 (62), 55 (30)	L_wax_, F_wax_, 4L, 4F
Fernenol	26.32	426 (3), 393 (20), 331 (12), 255 (14), 241 (35), 229 (10), 189 (10), 159 (11), 119 (25), 95 (52), 73 (100), 43 (64)	L_wax_, F_wax_, 1L, 1F
Unknown phytosterol III	26.78	440 (2), 422 (8), 129 (22), 121 (24), 119 (26), 107 (34), 95 (54), 73 (86), 69 (100), 55 (80)	L_wax_, F_wax_, 5L, 5F
Taraxasterol	27.15	426 (3), 408 (7), 189 (53), 175 (12), 161 (14), 147 (22), 129 (49), 121 (58), 109 (100), 95 (86), 73 (97), 55 (34)	L_wax_, F_wax_, 5L, 5F
Erythrodiol	27.33	442 (4), 216 (65), 203 (29), 119 (15), 95 (32), 75 (42), 73 (100), 55 (16)	L_wax_, F_wax_, 3L, 3F
Friedelin	27.51	426 (4), 163 (12), 149 (8), 123 (30), 109 (40), 95 (64), 81 (65), 69 (100), 55 (92)	F_wax_, 6F
Uvaol	27.76	442 (3), 216 (44), 203 (44), 188 (27), 133 (31), 73 (100), 55 (16)	L_wax_, F_wax_, 3L, 3F
Betulin	27.98	442 (4), 189 (13), 147 (10), 95 (26), 75 (39), 73 (100), 55 (14)	L_wax_, F_wax_, 2L, 2F
Oleanolic acid	28.19	482 (12), 456 (3), 320 (15), 203 (64), 189 (25), 129 (30), 119 (18), 73 (100), 55 (9)	L_wax_, F_wax_, 2L, 2F
Betulinic acid	28.34	456 (3), 320 (5), 203 (9), 189 (16), 129 (30), 73 (100), 55 (11)	L_wax_, F_wax_, 1L, 1F
Ursolic acid	28.77	482 (12), 456 (3), 320 (47), 203 (76), 189 (29), 133 (57), 73 (100), 55 (10)	L_wax_, F_wax_, 2L, 2F
Unknown derivative of triterpenoid acid I	29.21	452 (4), 320 (13), 203 (100), 189 (16), 148 (35), 133 (45), 119 (5), 75 (70), 73 (44), 69 (7)	L_wax_, F_wax_, 1L, 1F
Unknown neutral triterpenoid II	29.55	424 (3), 322 (9), 219 (8), 189 (20), 122 (16), 103 (35), 95 (28), 75 (37), 73 (100), 69 (15), 55 (12)	F_wax_, 3F
Unknown derivative of triterpenoid acid II	29.62	468 (3), 320 (15), 203 (50), 189 (16), 148 (40), 133 (45), 119 (17), 75 (25), 73 (100), 55 (8)	L_wax_, F_wax_, 1L, 1F
Unknown derivative of triterpenoid acid III	29.78	452 (6), 426 (3), 411 (3), 320 (12), 203 (75), 133 (35), 119 (18), 105 (13), 95 (15), 73 (100), 55 (9), 43 (58)	F_wax_, 3F
Maslinic acid	29.91	472 (3), 320 (9), 203 (22), 189 (9), 133 (12), 147 (39), 73 (100), 55 (6)	L_wax_, F_wax_, 1L, 1F
Corosolic acid	30.27	472 (3), 320 (12), 203 (22), 189 (6), 147 (42), 133 (23), 73 (100), 55 (8)	L_wax_, F_wax_, 1L, 1F

## Data Availability

All datasets generated for this study are included in the article.

## Supplementary Materials

The following supporting information can be downloaded at: https://www.mdpi.com/article/10.3390/antiox12020465/s1, Table S1: Summary of phytochemical features of obtained fractions and average cytotoxicity towards different cancer cell lines.
