# Supplementary material for: Fractionation and Characterization of Triterpenoids from Vaccinium vitis-idaea L. Cuticular Waxes and Their Potential as Anticancer Agents
Source: Antioxidants (Basel). 2023 Feb 12;12(2):465. doi: 10.3390/antiox12020465 (PMC9952570; doi:10.3390/antiox12020465)
Supplement: Supplementary file 1 [file antioxidants-12-00465-s001.zip › antioxidants-2146056-supplementary.pdf]

Table S1. Summary of phytochemical features of obtained fractions and average cytotoxicity towards different cancer cell lines.

Sample	Principal Triterpenoids (Type of Parent Skeleton)	Minor Triterpenoids (Type of Parent Skeleton)	Cytotoxicity		
			HT-29	IGR39	CaKi-1
F_{wax}	Crude cuticular wax extract of lingonberry fruits		+++	++	++
1F	Fernenol (Lupane type)	Corosolic acid (Ursane type)<br>Maslinic acid (Oleane type)<br>Betulinic acid (Lupane type)<br>2 unknown derivatives of triterpenoid acids<br>1 unknown neutral triterpenoid	+++	++	++
2F	Oleanolic acid (Oleane type)<br>Ursolic acid (Ursane type)<br>ratio of 1:2.2	Betulin (Lupane type)	+++	+++	+++
3F	Erythrodiol (Oleane type)<br>Uvaol (Ursane type)<br>ratio 1:4.0	1 unknown neutral triterpenoid<br>1 unknown derivative of triterpenoid acid	-	-	-
4F	Lupeol (Lupane type)	—	+	-	-
5F	β-Sitosterol (Stigmastane type)	β-Amyrin (Oleane type),<br>Taraxasterol (Taraxastane type)<br>Lanosterol (Stigmastane type)	+	+	++
6F	α-Amyrin (Ursane type)	Friedelin (Friedelane type)<br>β-Sitosterol (Stigmastane type)<br>2 unknown phytosterols	-	-	-
L_{wax}	Crude cuticular wax extract of lingonberry leaves		-	-	++
1L	Fernenol (Lupane type)	Corosolic acid (Ursane type)<br>Maslinic acid (Oleane type)<br>Betulinic acid (Lupane type)<br>2 unknown triterpenoid<br>1 unknown neutral triterpenoid	++	++	++
2L	Oleanolic acid (Oleane type)<br>Ursolic acid (Ursane type)<br>ratio of 1:3.7	Betulin (Lupane type)	+++	+++	+++
3L	Erythrodiol (Oleane type)<br>Uvaol (Ursane type)<br>ratio of 1:6.7	—	+++	++	+++
4L	Lupeol (Lupane type)	Lanosterol (Stigmastane type)<br>1 unknown phytosterols	+	-	-
5L	β-Amyrin (Oleane type)	β-Sitosterol (Stigmastane type)<br>Taraxasterol (Taraxastane type)<br>Lanosterol (Stigmastane type)<br>2 unknown phytosterols	-	-	++
6L	α-Amyrin (Ursane type)	—	+	-	-

Note: +++ cancer cells viability reduced more than 70%; ++ viability reduced by 40–70%, + viability reduced by 20–40%, - viability reduced less than 20%. Schematic results are presented based on average values of both tested concentration levels (12.5 and 25.0 μg/μL) and are only orientational.
